# Gendered Structural Inequality and Folk Illness Among New Mothers: An Autoethnography of *Shutika*

**DOI:** 10.1177/00469580261468781

**Published:** 2026-07-13

**Authors:** Shuma Banik, Bipin Adhikari

**Affiliations:** 1 Pandemic Sciences Institute, Nuffield Department of Medicine, 6396 University of Oxford, Oxford, United Kingdom; 2 Mahidol Oxford Tropical Medicine Research Unit, Bangkok, Thailand

**Keywords:** auto-ethnography, anthropology, cultural, culture-bound syndromes, medicine, traditional medicine, social determinants of health, healthcare disparities, developing countries, global health, structural injustice

## Abstract

**Introduction:**

This article explores *Shutika*, a folk illness from the Barak Valley region in India, through both emic and etic perspectives. *Shutika* lacks a biomedical equivalent but carries deep cultural significance, reflecting how communities interpret suffering and healing. Situating *Shutika* within broader South Asian and global health discourses underscores the importance of recognising such illnesses as expressions of socio-cultural and political realities.

**Methods:**

An autoethnographic approach was employed to capture the lived experience of *Shutika*. The emic perspective is conveyed through the author’s narrative as a daughter observing her mother’s illness, providing insight into local interpretations of causality, diagnosis, and cure. The co-author’s etic perspective contextualises *Shutika* within medical anthropology and global health.

**Results:**

The emic and the etic account reveals *Shutika* as both a physical and social affliction rooted in gendered expectations and structural inequality of traditional interpretation of illness.Traditional healing practices offered meaning and comfort beyond biomedical explanation.

**Discussion:**

The paper highlights the need for medical pluralism and collaboration between biomedical and traditional systems. *Shutika* emerges as a symbol of marginalised knowledge systems, calling for epistemic flexibility and culturally grounded approaches to global health.

## Introduction

*Shutika* is a culturally specific folk illness,^1^ its name derived from the Bengali word ‘*shuta*’, meaning “thread”.^2^ For many, these conditions persist for months or even years, becoming a lifelong physical and emotional suffering. Typically affecting new mothers, *Shutika* presents with a wide range of symptoms including diarrhoea, depression, severe weight loss, nausea, and prolonged weakness. *Shutika* exemplifies the anthropological concept of somatisation^3^ —the physical manifestation of psychological or social distress. This framework is particularly relevant for understanding the experiences of women from vulnerable communities, where social suffering, economic hardship, and gendered expectations often become embodied as illness. *Shutika* fulfils an entity proposed by Hofmann as a tri-modal concept of illness (how an individual perceives the condition), sickness (how society expects and labels the condition), and disease (a biomedical condition attributing condition).^4,5^

In the absence of biomedical recognition, treatment of *Shutika* typically involves local remedies—most notably, the blood from a local bird called *ghu-ghu (Spotted Dove in English*.

Mothers and their families generally consult traditional healers or elder women within the household, adhering to the cultural and traditional knowledge that existed in these low-income communities for centuries. *Shutika* is thus embedded in a traditional health system and is often viewed as an expected consequence of childbirth among women in these communities. *Shutika* therefore occupies a social space accommodable within a broader context of medical pluralism, where local health beliefs and practices operate alongside and sometimes contrarily with biomedical systems.

Shutika is experiences by mothers after childbirth in parts of lower Assam and Bangladesh,^1^ particularly among low-income, Bengali-speaking communities, indicating that its prevalence is closely tied to marginalised social contexts. Most existing literature on *Shutika* focuses on Bangladesh, while its occurrence within India is largely confined to the Barak Valley region of Assam which is home to marginalised Bengali-speaking populations with histories of migration and displacement from Bangladesh.^6^ In this region, ‘maternal behaviour’ is not limited to caregiving or reproductive roles but is a culturally prescribed set of practices through which women embody ideals of moral virtue and domestic order. It encompasses a range of domestic and ritual activities, such as observing specific times for bodily care, ensuring food is cooked and served punctually, maintaining ritual purity in the kitchen, and avoiding rest during the day. These practices are rooted in local moral economies that link a woman’s discipline and devotion to the well-being of her household. Consequently, failure to conform to these expectations is not merely perceived as personal negligence but as a moral transgression capable of disrupting household harmony and exposing the woman to afflictions such as Shutika. Despite this, the region and the condition has received minimal academic attention,^7^ underscoring the urgent need to examine *Shutika* within India’s and Barak Valley’s sociao-cultural landscape. This study seeks to address this gap by examining the socio-cultural, economic, and political dimensions of *Shutika* in the Barak Valley region of Assam, with a particular focus on Karimganj district which ranks the third poorest districts in Assam (Human Development Report, Assam, 2014), drawing from auto-ethnographical^7^ methods blending with concurrent literature.This paper will explore the intersections of poverty, caste, gender, migration histories, and local health-seeking behaviours influence the perception, treatment, and experience of this illness by employing emic and etic approaches by grounding the illness from insiders’ perspectives formed by lived experience and drawing from the extant scholarship on similar conditions across the globe. The emic and etic perspectives represent complementary approaches in anthropological research. The emic perspective seeks to understand experiences and meanings from the viewpoint of participants within their cultural context, whereas the etic perspective applies external analytical frameworks to enable objective analysis and comparison across settings. Integrating both approaches can provide a more comprehensive understanding of social and behavioural phenomena.^8^In doing so, we seek to contribute to broader debates on how bodies are not only biological but also social and political artefacts, shaped by systems of marginalisation and moral judgement.

## Methods

This article draws upon an autoethnographic methodology^9^ to explore and document the lived experience of the first author’s mother, who suffered from *Shutika*, including the experiential and embodied knowledge associated with this culturally bound folk illness, which does not have a direct biomedical equivalent. Autoethnography is a qualitative research approach that combines autobiography and ethnography in order to analyse personal experience within its broader cultural, social, and political contexts. It treats lived experience as a legitimate form of evidence through which cultural phenomena can be critically examined. The method has been increasingly employed across health and social science disciplines because it allows for the production of deeply personal yet analytically igorous narratives that remain contextually grounded while also engaging in critical cultural interpretation.^10^

As Pathak argues, autoethnography can function as a decolonial methodology that enables individuals embedded within specific cultural contexts to articulate their own stories and represent embodied forms of knowledge that are often marginalised within dominant academic discourse. In the case of *Shutika*, this methodological choice permits the reporting of a local folk illness from an insider perspective, thereby resisting its reduction into a purely Western pathological biomedical framework.^11^ Rather than translating *Shutika* into an already established diagnostic category, autoethnography allows the illness to be understood through its own cultural logic. Employing this method in the study of *Shutika* creates space for epistemic and pedagogical transformation by foregrounding lived experience as analytically valuable rather than anecdotal. As first author, I selected this methodology for two principal reasons. First, autoethnography offered a culturally appropriate mode of inquiry capable of capturing dimensions of suffering, care, and moral expectation that conventional positivist research designs often overlook or exclude. Second, it allowed me to honour and preserve the nuanced personal histories embedded in my mother’s experience, histories that risk being flattened when translated solely into biomedical or statistical categories. My mother’s diary, which consists of thirty single page handwritten entries, served as the primary data source. These entries document her deteriorating health following childbirth, the absence of sustained formal medical care, her interactions with indigenous healers, and her reflections on being diagnosed with *Shutika.* In addition to the diary, I engaged extended reflective conversations with my mother to better understand the broader social, familial, and cultural dynamics surrounding her experience of illness. My embeddedness within the same socio cultural environment shaped this research process. Shared language, everyday practices, moral expectations, and my position as both daughter and researcher enabled me to interpret her narrative with cultural fluency. At the same time, this proximity required careful reflexivity. As both researcher and daughter, I remained attentive to the intimate power dynamics shaping this work. I treated my mother’s diary not as neutral data but as a co constructed narrative emerging from relational trust. Reflexive journaling accompanied the analytical process in order to examine how my emotional proximity, memories, and disciplinary training influenced interpretation. To minimise the possibility of overshadowing her voice, I foregrounded her words and allowed her account to guide the structure and tone of the analysis.

My mother’s experience of *Shutika* directly shaped the writing of this manuscript. Revisiting her illness produced an emotional closeness that strengthened my commitment to presenting *Shutika* through its cultural and relational logic rather than through a reductive biomedical frame. Her narrative determined the pacing, sequence, and thematic focus of the autoethnography. The writing process thus became both analytical and relational, grounded in care, cultural fidelity, and respect for her lived experience. Data analysis followed a multi stage process. First, I translated the diary entries and conversation transcripts from Bengali to English. Second, I organised the material thematically by identifying recurring patterns, metaphors, and emotional expressions. This thematic analysis was supported by reflexive writing that contextualised my mother’s socio economic background and examined how her positionality as a woman from a marginalised community intersected with her experience of suffering. The analysis surfaced multiple interconnected themes, including gendered expectations, fear, guilt, shame, moral pressure, bodily exhaustion, and social isolation. After identifying these themes, I returned to my mother to discuss the interpretations. This act functioned as both an ethical safeguard and an epistemological checkpoints. It allowed me to verify that the analysis did not distort the cultural meaning of Shutika or impose external theoretical assumptions onto her account. Her feedback and affirmation grounded the findings in lived experience while recognising that individual narratives, though situated, hold significant epistemic value. In the results section, I employ a second person narrative to recount my mother’s journey through the lens of *Shutika*, beginning with childbirth and breastfeeding, moving through illness recognition and ritual treatment, and concluding with gradual recovery. The narrative unfolds chronologically in order to immerse readers in the embodied temporality of the illness. This storytelling approach humanises the experience while encouraging readers to critically reflect on how cultural norms, gendered expectations, and health systems shape interpretations of suffering. In doing so, the article opens space for dialogue between personal memory, collective cultural frameworks, and broader questions of health, gender, power, and care.

Although this study is primarily framed as an autoethnographic inquiry, the interpretive process also incorporated collaborative elements through ongoing dialogue, participant reflection, and co interpretation of diary narratives. In this sense, the study shares some features with collaborative autoethnography, where lived experience and analytic interpretation are jointly negotiated while remaining distinct in its focus on a single familial illness narrative rather than co authored experiential accounts.^12^

## Findings

Analysis is organised into two interconnected components. First, it presents the phenomenological account of Shutika as experienced and recorded by my mother in her personal diary and in subsequent oral narratives shared with me. Second, it offers a layered interpretation of the illness through both an emic perspective grounded in lived experience and an etic perspective that situates Shutika within broader theoretical debates on illness narratives, gendered embodiment, idioms of distress, and vernacular healing practices.

### My Mother’s Experience of Shutika

This section explores my mother’s embodied and social experience of Shutika. It highlights how cultural expectations, gendered moral frameworks, and socioeconomic vulnerability shaped her illness trajectory. It also examines the challenges she encountered while navigating a formal healthcare system that did not recognise her condition within biomedical categories.

### Bodily Experience of Illness

My mother’s daily journal entries reveal how the illness progressively limited her physical abilities and compromised her autonomy, particularly in carrying out everyday domestic responsibilities. Routine activities such as cooking, cleaning, climbing stairs, or breastfeeding became physically overwhelming. She repeatedly described feeling completely depleted. Her emotional and physical symptoms were deeply intertwined. Palpitations, breathlessness, gastrointestinal distress, and persistent anxiety rendered even simple movements exhausting. One entry reads, “I can’t climb the stairs. My heart is always racing.” In this account, the body becomes the primary site through which distress is communicated.

**Figure 1.**
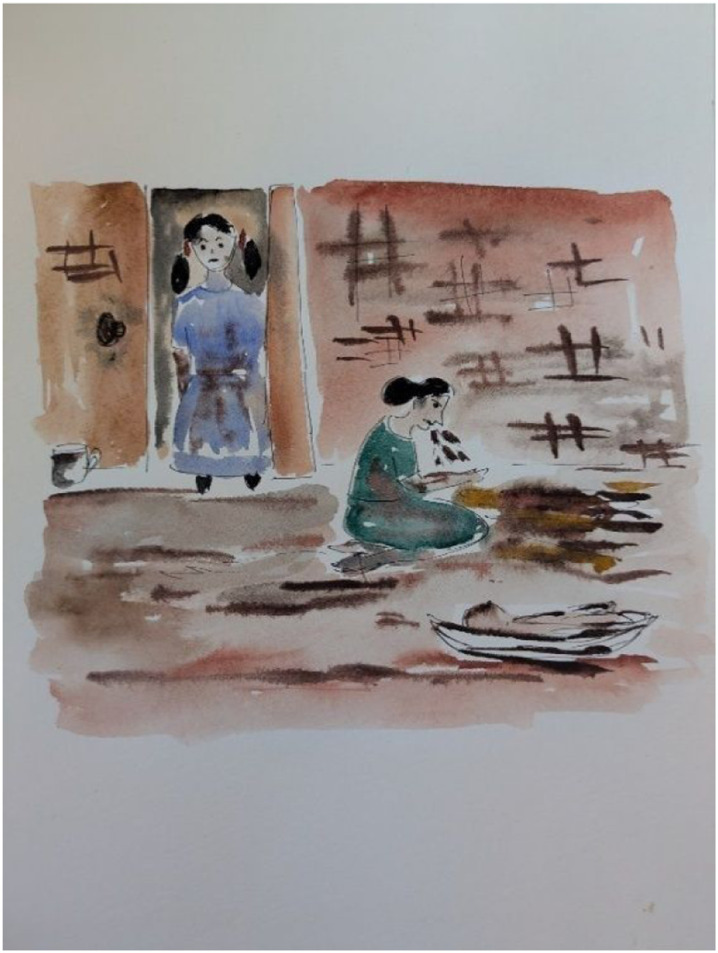
Mother on bathroom floor with her new born (Illustrated by 1st Author, Shuma Banik)

In another entry, she wrote:

“I feel I am failing as a mother. I don’t have anyone to hold my newborn when I go to the bathroom. Today, I put her in a cot in the washroom while I showered, and I don’t remember when I fell asleep on the floor. I woke up to her cries. I feel horrible that she has to see me like this. I want to get better, but I don’t know if I ever will.” Her words reveal more than physical weakness. They expose the moral burden of motherhood within her sociocultural context. The inability to perform maternal duties was not experienced simply as fatigue but as moral inadequacy. Her body became a site of meaning making where physical depletion was interpreted as failure to embody the ideal of the resilient and self-sacrificing mother as illustrated in Figure 1.

Her diary also reflects spiritual distress. She questioned God and expressed growing disbelief, signalling a rupture in her previously stable faith. At the same time, she expressed intense guilt over inadequate breast milk production, linking it directly to shame and perceived incompetence. These reflections demonstrate how biological processes were interpreted through moral and cultural expectations.

**Figure 2.**
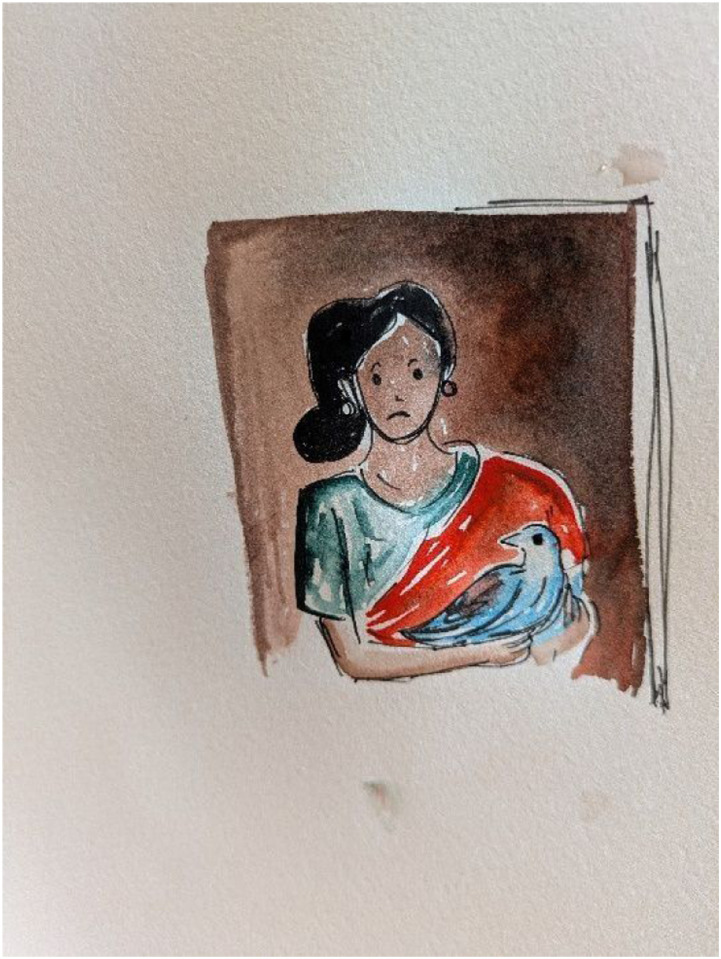
Distress of drinking the raw blood of the bird and smearing the remaining blood on the head (Illustrated by 1st Author, Shuma Banik)

Alongside emotional suffering, she documented stomach pain, diarrhoea, itchy skin, and extreme fatigue. She vividly described the distress she felt when required to drink the raw blood of the Ghu Ghu bird and smear the remaining blood on her head and forehead as illustrated in Figure 2.

Although undertaken as a culturally sanctioned remedy, the ritual was experienced as physically and emotionally disturbing.

While the ritual embodied hope for healing within indigenous medicine practices, it also illustrates how the body becomes the terrain upon which cultural meanings of cure and suffering are enacted. Despite the intensity of her pain, her tone often conveyed quiet resignation. Suffering appeared normalised. Her family framed endurance as a feminine virtue. Medical neglect was rationalised through the expectation that women must tolerate discomfort after childbirth. In this way, her body functioned simultaneously as a site of stigma, moral evaluation, and muted resistance.

### Social Experience of Illness

Illness experience is inseparable from its social context. *Shutika* emerges from the socio symbolic landscape of the Barak Valley, where postpartum vulnerability is interpreted through community narratives and shared moral frameworks rather than biomedical diagnostics.

*Shutika* does not align neatly with biomedical categories. Instead, its meaning and management are shaped by mothers, elderly women, community members, and indigenous healers. In the absence of clinical recognition, knowledge of the illness circulates through experiential testimony and intergenerational transmission. This vernacular medical epistemology is relational and cumulative, grounded in shared histories and affective memory.

**Figure 3.**
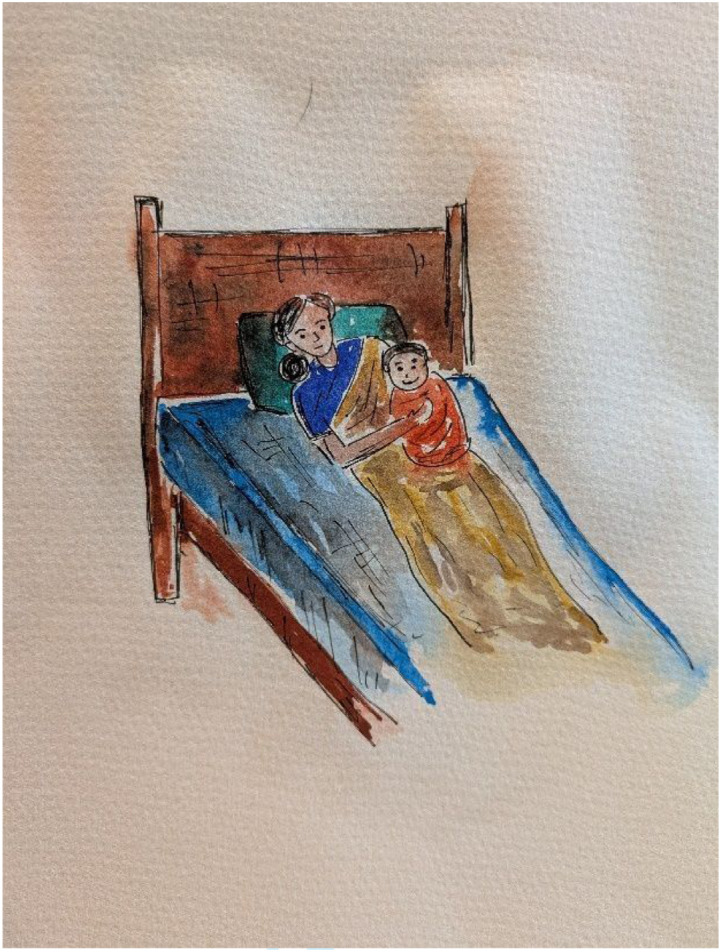
Everyday battle of dealing with “Am I a good mother?” (Illustrated by 1st Author Shuma Banik)

My mother’s journal reflects her entanglement within normative expectations of womanhood and questioning herself as a mother as illustrated in Figure 3. She wrote: Every mother goes through what I am going through. Why am I not able to bounce back to good health? Am I cursed? Everyone gets on with their lives. As women, we are supposed to face these realities and still embrace them. Why can’t I? I don’t know why women are always expected to be so perfect. I am tired.”

**Figure 4.**
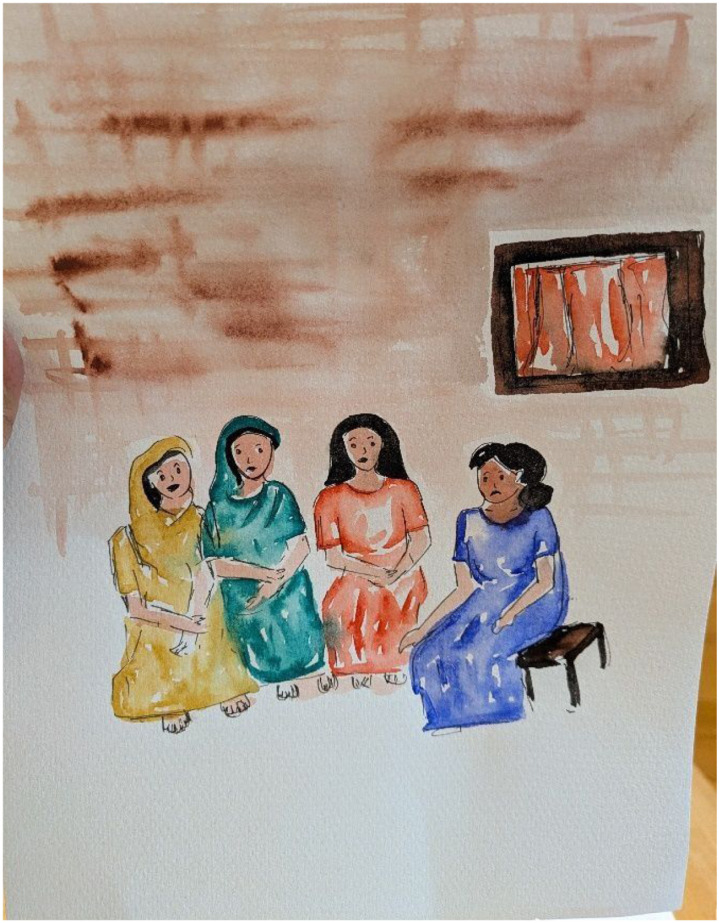
Women telling her that it is a natural progression to womanhood (Illustrated by 1st Author Shuma Banik)

Her reflections reveal the internalisation of social comparison and moral judgment. Community responses further illustrate the social framing of her suffering. She was often told, “Do not overthink. Take care of your family and children. Most women have it worse than you” as illustrated in Figure 4. While the diagnosis of *Shutika* provided a culturally recognised label that connected her to others with similar experiences, it simultaneously trivialised her distress. Recognition and minimisation coexisted. Over time, this cultural trivialisation led to withdrawal from social spaces. She reported being perceived as overly sensitive or dramatic. Unable to conform to the idealised image of the resilient mother, she entered a prolonged period of isolation, described as one of the most difficult years of her life.

Reflecting on my childhood, I remember witnessing my mother’s physical, emotional, and spiritual distress following the birth of my younger sister. At the time, I struggled to interpret her suffering. As a global health practitioner years later, I initially viewed her symptoms through a biomedical lens, considering postpartum depression as a possible diagnosis. However, through autoethnographic reflection and engagement with scholarship on folk illness, I came to understand that her suffering was situated within a culturally recognized category of folk sickness.^13^ This shift represents both change and continuity. The biomedical vocabulary became more familiar over time, yet the cultural logic of *Shutika* remained constant within the community. *Shutika* can be understood as rooted in gendered labour, socioeconomic precarity, and systemic neglect. It reflects a contextualised interpretation of postpartum vulnerability within low income households. Recognising this requires engagement with indigenous knowledge systems that generate, store, and transmit understanding through oral narratives, ritual practice, and embodied memory.

### Etic Perspective

From an etic standpoint*, Shutika* can be interpreted as an idiom of distress.^14^ Idioms of distress often emerge in settings where emotional expression is constrained and structural support is limited. In such contexts, the body becomes the medium through which social suffering is expressed. This aligns with the concept of protest of the body,^15^ wherein physical symptoms articulate unspoken social strain. *Shutika* legitimises women’s suffering within culturally sanctioned frameworks. It provides explanatory coherence and communal recognition. At the same time, it may reinforce expectations of endurance within patrilineal social structures that limit women’s autonomy and expressive freedom. What has persisted across time is the gendered burden of care and the moral expectation of maternal resilience. What has shifted is increasing awareness, particularly among younger generations and practitioners, of maternal mental health within global health discourse. However, in marginalised regions such as the Barak Valley, structural constraints and epistemic marginalisation continue to shape illness interpretation. Given its complex interplay of social, cultural, and economic determinants, *Shutika* demands sustained anthropological and global health inquiry that considers gender, politics, and systemic inequality alongside clinical concerns.

### Limitations

This study has several limitations. As an autoethnographic account centred on a single individual’s lived experience, the findings are not intended to be generalisable beyond the specific socio-cultural context examined. Although the use of contemporaneous diary entries strengthens credibility, the analysis remains interpretive and shaped by retrospective reflection, translation from Bengali to English, and the first author’s dual positionality as both daughter and researcher, which may introduce subjective bias despite reflexive safeguards and member checking. The absence of perspectives from other affected women, traditional healers, or biomedical practitioners limits triangulation and a more comprehensive understanding of how Shutika is negotiated across social and clinical spaces.

The questionnaire used in this study was not formally validated or pilot-tested prior to administration, which may limit the reliability and generalizability of the findings. Furthermore, the study does not undertake clinical assessment to determine overlap with biomedical diagnostic categories, as its primary aim is cultural and structural interpretation rather than nosological clarification. These limitations notwithstanding, the depth of contextual insight offered by autoethnography provides a valuable contribution to medical anthropology and global health scholarship on postpartum suffering in marginalised settings.

## Discussion

### Emic and Etic Perspective

This study approaches *Shutika* through both emic and etic lenses. The emic perspective is embodied in the author’s narrative, which draws on lived experience as a daughter who witnessed her mother’s suffering from Shutika. This insider account supplies a richly contextualised understanding of how diagnosis, causality, and treatment are interpreted locally. The etic perspective, contributed by the co author, situates *Shutika* within the wider South Asian region and academic literature on folk illnesses and lived experience.^13^ This external lens highlights the absence of a biomedical equivalent for *Shutika* and emphasizes that such conditions are not only pathophysiological phenomena but also expressions of social, cultural, and political reality. Folk illnesses like *Shutika* are at risk of being dismissed as superstition. This tendency is rooted in a colonial epistemology that delegitimises knowledge systems outside Western biomedicine. A sociocultural understanding of folk illnesses provides a comprehensive basis for designing effective therapeutic interventions. Integrating emic and etic frameworks enables a nuanced and holistic account that respects both lived experience and scholarly analysis and challenges the epistemic boundaries imposed by dominant medical paradigms.

### Autoethnography as a Method

Writing about *Shutika* through an autoethnographic lens is not merely a methodological choice but also a political and epistemological intervention. Autoethnography foregrounds insider voice and situated knowledge while placing those voices in dialogue with theory.^11,16^ In this study the author used diary material and relational memory to ground the narrative. Working with a co author enabled triangulation between situated knowledge and regional theoretical perspectives drawn from global health literature, feminist epistemologies, and decolonial scholarship.^17^ This collaboration sustained narrative fidelity while allowing critical engagement with established academic frameworks. The participant was not treated solely as a subject of analysis but also as an active contributor to interpretive meaning making. Recurrent discussions regarding the diary entries shaped thematic interpretation and strengthened reflexive engagement throughout the study.

### Epistemic Injustice and Knowledge Production

Shutika exemplifies epistemic injustice.^18^ The author’s mother experienced dismissal and normalisation of pain by family members and healthcare providers. Her experience was rarely recorded in medical documentation and lacked formal diagnostic recognition. Such omission is not incidental. It is linked to gender and socioeconomic position that shape which forms of knowledge are authorised within medical institutions. Recognising epistemic injustice requires exposing the mechanisms by which some forms of knowledge are marginalised and investing in research that documents and values local knowledge systems. In this context the phrase absence of evidence is not evidence of absence^19^ clarifies how methodological blind spots can be mistaken for clinical absence. Indigenous medicine practices are deeply embedded in community life. They provide interpretive frameworks and practical care that are relational and narrative based. Community based diagnoses and treatments are transmitted intergenerationally and remain. Resilient because they address social, spiritual, and material dimensions of suffering. Yet these practices are often undocumented and unacknowledged within formal biomedical discourse. Greater research investment in these systems would expand the evidence base and help bridge mistrust between communities and biomedical institutions.^20^

### Illness, Sickness, and Diseases

The World Health Organization defines health as a state of complete physical, mental, and social well being and not merely the absence of disease or infirmity.^21^ Applying the triad of disease, illness, and sickness clarifies *Shutika*’s positioning.^22^ Disease refers to biomedical pathology. Illness refers to embodied experience. Sickness refers to socially recognised suffering. Appreciating this distinction enables clinicians and researchers to engage with people from the perspective of experience as shaped by culture and socioeconomic background. This approach improves communication about health seeking behaviour and clarifies reasons for delayed or selective care. Lessons from conditions that carry stigma such as leprosy, tuberculosis, and HIV demonstrate that illness experience is shaped by social stereotypes, marginalisation, and ecnoomic background.^23-25^ These examples caution against reducing *Shutika* to an individual pathology and encourage attention to its social framing.

### Implications for Medical Pluralism

*Shutika* is not an isolated example. Other culturally embedded conditions such as Move San in Haiti^26^ and Susto in Latin America^27^ demonstrate how idioms of distress operate across contexts. Medical pluralism^28^ offers an inclusive approach by recognizing coexistence of multiple knowledge systems. A purely biomedical solution can be reductive.^29,30^

Collaboration between community based practitioners and biomedical providers can capitalise on existing resources and foster complementary care pathways.^31^ Respecting medical pluralism also helps bring forward folk illnesses that are often subdued in society, which may otherwise result in delayed treatment seeking and complications.^32,33^ Understanding *Shutika* within its cultural and socio political context enables interventions that address structural determinants alongside symptoms. Ultimately, what is required is epistemic flexibility,^34^ a willingness to engage social manifestations of suffering as legitimate forms of knowledge rather than dismissing them as peripheral to clinical care.

## Conclusion

*Shutika* reveals how postpartum suffering among marginalised women in the Barak Valley cannot be understood solely through biomedical categories. Through relational autoethnography, this study demonstrates that *Shutika* embodies gendered moral expectations, structural poverty, and epistemic marginalisation. The illness is not merely cluster of symptoms but a culturally mediated expression of distress shaped by social norms, domestic labour burdens, and limited institutional recognition. While elements of *Shutika* may overlap with biomedical constructs such as postpartum depression, reducing it to a diagnostic label risks obscuring its relational, moral, and political dimensions. Recognising *Shutika* through both emic and etic lenses underscores the importance of medical pluralism and epistemic flexibility in global health. Culturally grounded approachesthat engage indigenous knowledge systems alongside biomedical care are essential for addressing structural inequities and improving maternal health outcomes in marginalized settings.

## Data Availability

Due to the personal and sensitive nature of the diary material, the full dataset is not publicly available. Selected de-identified excerpts are included within the manuscript. Additional information may be available from the corresponding author upon reasonable request and with participant consent. All the data is available in the study article. Any other journal-specific required statements.*
